# Assessment of App-Based Versus Conventional Survey Modalities for Reproductive Health Research in India, South Africa, and the United States: Comparative Cross-Sectional Study

**DOI:** 10.2196/44705

**Published:** 2023-12-01

**Authors:** Amanda A Shea, Jonathan Thornburg, Virginia J Vitzthum

**Affiliations:** 1 Biowink GmbH Berlin Germany; 2 Department of Astronomy & Center for Spacetime Symmetries Indiana University Bloomington, IN United States; 3 Centre for Menstrual Cycle and Ovulation Research Division of Endocrinology/Medicine The University of British Columbia Vancouver, BC Canada; 4 Department of Anthropology Indiana University Bloomington, IN United States

**Keywords:** mobile health, mHealth, femtech, reproductive health, menstrual health, sexual health, survey modalities, menstrual tracking app, India, South Africa, United States, mobile phone

## Abstract

**Background:**

There is a widely acknowledged global need for more research on reproductive health (including contraception, menstrual health, sexuality, and maternal morbidities) and its impact on overall well-being. However, several factors—notably, high costs, considerable effort, and the sensitivity of these topics—impede the collection of the necessary data, especially in less accessible and lower-income populations. The burgeoning ownership of smartphones and growing use of menstrual tracking apps (MTAs) may present an opportunity to conduct reproductive health research with fewer impediments than those associated with conventional survey methods.

**Objective:**

The main objective was to ascertain the feasibility, potential usefulness, and limitations of conducting reproductive health research using a mainstream MTA.

**Methods:**

In each of the 3 countries, we evaluated questionnaire responses from (1) current users of an MTA (Clue) and (2) participants surveyed using conventional survey modalities (in-person interviews, SMS text messaging, and web-based questionnaires). We compared these responses with published data collected from large nationally representative benchmark samples (the United States Census and the Demographic and Health Surveys for South Africa and India).

**Results:**

Given a sufficiently large user base, app-distributed surveys were able to quickly capture large samples on par with other methods and at low cost, with the additional advantage of being able to deploy remotely and simultaneously across countries. In each country, neither the app nor the conventional modality sample emerged as a consistently closer match to the distributions of the demographic attributes and the patterns of contraceptive use reported for the respective benchmark sample. Despite efforts to obtain representative samples, the conventional modality samples sometimes over- and other times underrepresented some subgroups (eg, underrepresentation of married persons in the United States and overrepresentation of rural residents in India). In all 3 countries, app users were younger, more educated, more likely to be urban residents, and more likely to use nonhormonal rather than hormonal contraceptive methods compared with the respective national benchmark. App users, compared with the conventional modality samples, consistently reported being more comfortable discussing their menstrual periods with other persons (eg, family, friends, and health care providers), suggesting that MTA users may be more likely to respond truthfully to questions on sensitive or taboo health topics. The app samples’ consistency across countries regarding users’ demographic profiles, contraceptive choices, and personal attitudes toward menstruation supports the validity of making cross-country comparisons of survey findings for a given app’s users.

**Conclusions:**

MTAs such as Clue can provide a quick, scalable, and cost-effective method for collecting health data, including on sensitive topics, across a wide variety of settings and countries. With expanding global access to technology and the increasing use of these tools, consumer MTAs can be a viable survey modality to strengthen reproductive health research.

## Introduction

### Background

The reproductive system is an integral component of bodily functioning and overall well-being. Reproductive health encompasses the proper functioning of the reproductive system at all developmental stages throughout life and includes the ability to have a satisfying and safe sex life and the freedom to decide whether and how to reproduce [1,2]. It is widely acknowledged by governments and international organizations, health care researchers and providers, and the general population that there is a pressing global need to improve reproductive health for all people. Accurate comprehensive data are invaluable for effectively addressing this challenge. However, in most countries, there are surprisingly few data on the reproductive health of the population or on the availability of health services and other resources necessary to achieve population-wide satisfactory reproductive health. The acquisition of such data can be daunting.

A crucial component of efforts to reduce maternal and infant mortality and morbidity over the past few decades has been and is the collection of nationally representative health data, often conducted by a nation’s government, sometimes in collaboration with nongovernmental agencies (eg, the Demographic and Health Survey [DHS] Program [3]). These data are used to assess a population’s current health status, evaluate the impacts of programs, and inform future efforts to improve health. Careful planning, technically sophisticated statistical sampling, and pretested standardized questionnaires (customized to a population’s cultural and socioeconomic attributes) administered in face-to-face interviews by trained staff underpin the reliability and representativeness of DHS data [4]. Therefore, it was and is possible to accurately monitor the gains and setbacks in maternal and infant health over several decades even in the poorest populations.

However, DHS and other nationally representative studies require considerable effort, expense, and time, all of which limit the coverage of different health issues and preclude frequent surveys or modifying questionnaires in response to unanticipated developments (eg, new diseases and novel trends affecting population health). For example, during the COVID-19 pandemic, rapid changes in access to contraceptives and health care services occurring at different times and to different degrees across locations were difficult to document and unlikely to be captured in a country’s DHS survey. Such unanticipated and rapid shifts in health status and services can fall under the radar of surveys and censuses that take a year or more to organize and are typically separated by several years.

Smaller-scale targeted surveys, which are more rapidly and easily deployed, are a widely used approach for gathering data that complement those from national studies. Small-scale surveys can gather useful subsets of the same data types as national studies, target a single subpopulation, or explore new health issues. There are several conventional modalities commonly used in such surveys, including one-on-one interviews (in person or by telephone), mailed questionnaires to be completed and returned by a respondent, web-based questionnaires distributed via social media and other websites, and SMS text messages to cell phones.

Each of these conventional modalities has its own strengths and weaknesses in terms of representativeness, generalizability, costs, sample size, and collection speed [5,6]. For example, meetings between a respondent and trained personnel who administer a questionnaire may be difficult to arrange. Spontaneous telephone calls may be rebuffed, especially if the potential respondent is busy. However, if respondents are compensated (as is typically the case for commercial survey providers), completion rates can be very good, although the costs can be high.

Using smartphones and associated mobile apps for data collection can be a more rapid, cost-effective, and scalable modality than other conventional modality surveys (CMSs). The rapid growth of smartphone use makes it particularly suitable for collecting data from a broad swath of a population. Although smartphone ownership remains more widespread in higher-income countries and among younger, more educated people, as well as among men as compared with women, smartphone ownership is increasing across the board, with these gaps narrowing over the past decade [7]. Smartphone ownership is now expected to expand to more than two-thirds of the global population by 2025 [7].

With the increasing ubiquity of mobile devices, there has been a parallel increase in their use for public health and medical practice, referred to as mobile health (mHealth), which has been used in various settings from medication adherence in clinical practice to behavior change interventions in large populations [8]. Among the advantages over most CMSs, mobile app data collection can be implemented remotely and completed at the convenience of the respondents. Thus, participants do not need to live near or visit a research site to take part, increasing the ability to include those limited by travel burdens or even those who simply want the convenience of participating whenever and wherever they prefer [9]. Similarly, researchers do not need to travel to conduct data collection. These various advantages reduce staff and participant costs, thereby reducing research expenses. Owing to the widespread use of smartphones, researchers can more easily reach a larger and potentially more diverse population, yielding greater sample sizes and more generalizable results. The possibility of continued direct communication with participants via mobile apps also makes this modality suitable for follow-up or for use in longitudinal studies. App-based data collection may also provide respondents with greater anonymity and increased privacy and comfort, which, in turn, could improve respondent accuracy even for culturally taboo topics. In contrast, completion rates may be low and data quality may be poor if respondents are not motivated to provide truthful, complete answers to questions.

Theoretically, almost any mainstream smartphone app with a large user base can serve as a conduit to a pool of potential survey respondents. However, health apps are likely to be a particularly productive choice for reaching people who are motivated to provide truthful answers to questions regarding their own health as these users have demonstrated an interest in the topic. As menstruation is a practically universal experience in female individuals from puberty to menopause, we hypothesized that menstrual tracking apps (MTAs), which are already widely used by both adolescent girls and adult women [10-12], are exceptionally well positioned for collecting reproductive health data from this segment of the population. Furthermore, menstrual health is a key component of overall health as well as reproductive health [13-15]. Proper functioning of the menstrual cycle is obviously essential to fertility and is a key factor in contraceptive decision-making [16]. Moreover, menstrual cycling affects nearly all aspects of female health, including immune function [17,18], mental health [19,20], cardiovascular health [21], and bone health [22]. Many MTAs provide support for a range of use cases, including menstruation, contraceptive use, conception, pregnancy, and perimenopause, with the ability to track a broad range of experiences such as periods, energy, mood, pain, sex, sleep, exercise, illness, and medications. Clearly, there is an expansive array of health topics that could be addressed by incorporating MTAs into research designs.

However, there is currently little information on the feasibility of conducting surveys via MTAs or information on the respondent characteristics of those who have been recruited using these platforms.

### Objectives

Our study aimed to help address this research gap. Specifically, in each of the 3 countries (India, South Africa, and the United States), we evaluated the comparability of questionnaire responses collected using a CMS and an MTA to highly reliable benchmark data collected by the respective national governments. The study questionnaire included sections on individual demographic attributes and current contraceptive use (topics that are typically included in national-level reproductive health surveys) and a section on the respondents’ attitudes regarding menstrual bleeding (which potentially differ between MTA users and the general population and which has emerged as a significant but understudied factor affecting contraceptive choice [16]). We also assessed differences in performance, including ease of use and ability to achieve desired sample sizes, between the CMS and the MTA.

## Methods

Clue is a smartphone app for tracking menstrual bleeding and other aspects of health (eg, pain, sleep, and emotions). Owing to its large global user base (10 million active users worldwide), we hypothesized that the Clue app [23] would be a suitable choice for research in different cultural and demographic contexts.

### Ethical Considerations

This project underwent ethics review in each of the 3 study countries. It was deemed exempt by the Western Institutional Review Board in the United States, approved by the Sigma institutional review board in India (10008/IRB/20-21), and approved by the Research Ethics Committee at the University of Pretoria in South Africa (reference 05079421 [HUM042/0620]). In accordance with each CMS provider’s policy and the approved protocol, CMS respondents received nonmonetary gifts (such as reward points and phone top-ups) but did not receive direct monetary compensation. App-based survey participants were not paid or otherwise compensated for taking part in this study.

Only persons aged 18 to 45 years were eligible to participate in the study. All prospective participants were provided with study information, including the study goals, how study data would be processed and stored (and that survey responses would be archived for other researchers to use to answer additional research questions), and the contact information of the researchers for any questions or concerns. All participation was completely voluntary, and informed consent was obtained before participants could begin the survey. Except for Clue users in the United States, where app survey data were combined with an individual’s app-tracked data (with the consent of the participant), all surveys were taken completely anonymously.

### Analytical Strategy

In each country, we compared data that were collected using different modalities and that, by the nature of their data collection protocols, represented different segments of each country’s population:

1. A questionnaire sent to a random sample of current Clue app users
2. A shortened version of the same questionnaire distributed by commercial survey companies using conventional modalities (in-person interviews, web-based questionnaires, and mobile phone SMS text message surveys)
3. Publicly available, nationally representative demographic databases (these data provided a reliable benchmark against which to assess the demographic composition of the app and CMS samples)

### Study Countries

In total, 3 countries were selected for this study based on the following considerations:

1. Presence of country-specific, recent, publicly available, and nationally representative demographic survey data
2. Sufficiently large Clue user base such that, given typical user click-through rates for app-based surveys, an adequate sample size for the intended analyses was likely to be achievable
3. Representation from 3 distinct global regions
4. Representation of different population profiles of contraceptive use

The United States, India, and South Africa met these criteria. These 3 countries were also selected as India was a Family Planning 2020 target country [24], South Africa had greater use of long-acting injectables than either the United States or India, and previous studies have reported that cultural norms and beliefs regarding menstrual bleeding differ greatly between these populations [25-28].

### Questionnaire Development and Testing

Respondents were asked questions about their demographic profile, current and past contraceptive use (if any), and attitudes and preferences regarding menstrual bleeding. The number of questions varied slightly across countries to accommodate local differences in demographic categories and the limitations of the different modalities. All versions of the study questionnaire are provided with the archived data.

For each country, the goal was to collect data from a CMS participant sample that would resemble a sample that could have been recruited using standard research methods in that specific setting. This approach allowed for the country-specific comparison of CMS, app, and benchmark data. However, because of the necessary differences in recruitment criteria for the CMS (a reflection of already known sociodemographic differences between the countries), the study design was not specifically intended to collect CMS data that were directly comparable across all 3 countries. In contrast, befitting the lack of information before the study on the attributes of the app population, data collection from the app samples did not use targeting strategies or quotas. This approach for this group allowed for an evaluation of the distribution of the characteristics of the current app user base.

The study questionnaire was first developed for English speakers in the United States and tested via in-person interviews and a web-based testing platform, UserTesting. A native Spanish consultant followed the same protocols for testing the questionnaire with Spanish speakers in the United States. Subsequent user testing in South Africa and India was conducted under the direct guidance of in-country experienced research consultants. The country-specific questionnaires were extensively tested for local suitability (eg, income and ethnicity categories) and comprehension (eg, locally colloquial language for types of contraceptives and for attitudes regarding menstrual bleeding).

### App-Based Data Collection

Data were collected from November 9 to 29, 2020, in the United States and from November 12, 2020, to February 17, 2021, in India and South Africa. Invitations with a link to the questionnaire were distributed via in-app message in English to randomly selected Clue users in each of the 3 study countries and also in Spanish to those users in the United States whose phone language was set to Spanish.

As people responding to app-based questionnaires may enter false or impossible answers (perhaps to pass the time or for personal amusement), it was explained to the app users at the start that the survey was for research purposes. As a final quality check, the respondent was asked to answer *yes* or *no* to the following statement: “I took the survey seriously.” Only those who answered *yes* were included in the analyses.

The target sample sizes for each country reflected the country-specific Clue app user base and likely completion rates, and the sample size needed for the planned analyses. For India and South Africa, the target sample sizes were set at 2500 completed questionnaires each, equal to the target set for the CMSs described in the following section. Taking advantage of the larger Clue user base in the United States, this target sample size was set at 10,000 completed questionnaires so as to provide adequate statistical power for future subgroup analyses. Data collection ended when the target was reached or when the daily response rate had decreased to near 0.

### CMS Data Collection

A commercial third-party research company in each country collected the questionnaire data using various CMSs [29-34]. The specific approach used in each country was selected based on cost, ease of use under local conditions, and likelihood of accessing low-income participants. The questionnaires were suitably modified to fit the data collection method. The target was 2500 completed surveys for each country; this sample size would be both sufficient for the planned analyses and affordable.

The CMSs used were (1) web-based questionnaires delivered to a research panel representative of the broader population in the United States; (2) in-person interviews administered by health workers at the respondents’ homes in the states of Bihar and Uttar Pradesh in India; and (3) questionnaires administered via SMS text messaging to mobile phones nationally in South Africa, which were supplemented with tablet surveys administered in person at respondents’ homes to have a better representation of the lowest socioeconomic group.

Multimedia Appendix 1 provides information on the commercial survey providers and the details of each country’s CMS.

### Nationally Representative Demographic Data

Demographic data collected from the app surveys and CMSs were subsequently compared with publicly available benchmark data (sources discussed in this section) to assess the locale-specific representativeness of the respondent samples. The CMS and app surveys included only persons aged 18 to 45 years. Published statistical analyses for the benchmark samples are, for some variables, based on different age ranges and, in the case of sensitive data (eg, contraceptive use), may be restricted to persons who are currently married. The specific characteristics of the benchmark and other modality samples are provided in the country-specific tables in the *Results* section.

When necessary for correct cross-modality comparison, the reported data were weighted, and this was noted in the tables when applicable. For example, the South African DHS reports the percentage of the entire population (of all ages) for each of the age bins listed in the table reporting South African participant demographics in the *Results* section. The column for the app-based survey reports the percentage of the *sample* for each of these age bins. To compare the 2 age distributions, each age bin for the DHS sample was weighted to the summed percentage for the bins from the ages of 18 to 45 years. Benchmarks that only reported a variable’s distribution for those aged 15 to 49 years were not weighted.

The selected benchmarks for the United States were the US 2020 census [35] for the demographic variables and the US Centers for Disease Control and Prevention National Survey of Family Growth (approximately N=6000; 2017-2019) for contraceptive use variables [36].

For the India and South Africa comparisons, the selected benchmarks for demographic and contraceptive use variables were the most recently available DHS [3] data in each country (2016 for South Africa, 2019-2021 for India and Bihar, and 2015-2016 for Uttar Pradesh). All DHS data were collected via in-person interviews. The sampling method included a probability-based multistage stratified random cluster sample of households nationwide with poststratification weights applied to correct for minor selection and nonresponse biases. The total DHS sample sizes for women aged 18 to 45 years were approximately 8514 in South Africa and 521,300 in India. As approximately half of the Indian CMS sample came from each of 2 Indian states (Bihar and Uttar Pradesh), the benchmark for “Current Contraceptive Use” in the CMS sample was a 50/50 weighted average of the DHS-reported findings for these 2 states. The Indian app-based survey was nationwide; therefore, the benchmark for this sample was India’s national DHS report.

## Results

### Recruitment

Table 1 reports recruitment statistics for the app-based survey in each country.

**Table 1:** App-based subsamples from initial contact to completed data collection. Total number of survey invitations (in-app messages and push notifications) sent to app users, user click-through rates, and final sample sizes eligible for the analyses in this report.

Country	In-app message (number of users)	Push notifications (number of users)	User click-through rate (of those contacted), n/N (%)	Ineligible users (screened out by survey)	Completed and eligible for these analyses
India	104,890	66,243	10,392/171,133^a^ (6.1)	2680	1246
South Africa	30,720	19,358	6,151/50,078^a^ (12.3)	1049	879
United States	438,183	N/A^b^	29,187/438,183^a^ (6.7)	7776	10,244

^a^Denominator = in-app message + push notifications.

^b^N/A: not applicable.

### Demographic Characteristics of the Study Samples

#### United States

Of the 2500 respondents who completed the CMS (ie, Dynata web-based questionnaire [29]), 2464 (98.56%) answered questions regarding demographic attributes, contraceptive method use, and menstrual bleeding attitudes. A total of 10,244 app respondents answered these same questions and also explicitly affirmed that they had taken the survey seriously.

Table 2 presents the distribution of respondents in the United States by age, educational level, marital status, and residence locale, and Table 3 presents race or ethnicity of women aged 18 to 45 years from each of the 3 data collection methods: conventional web-based survey, app-based survey, and the 2020 US census (the benchmark) [35].

By design, the CMS sample was intended to be a good demographic match for the benchmark. As expected, the distribution of this survey sample’s age categories was within 3% of that of the benchmark except for overrepresentation of the oldest age group. The app survey sample was within 3% of the benchmark in the ages of 18 to 19 years and 30 to 34 years but substantially overrepresented those aged 20 to 29 years and underrepresented those aged 35 to 45 years.

Education categories in the CMS sample were within 2% of the benchmark. The app sample was also within 2% of the benchmark for the proportion who held high school degrees but overrepresented those with higher education by 9% (7340/10,244, 71.65%). There were very few app respondents who had not completed high school. The proportion of rural participants in both the CMS and app survey was within 3% of the benchmark. Both surveys underrepresented suburban residents; the app survey substantially overrepresented urban residents. The proportion of women in the United States who had never married at the age of 33 years (the median age of the conventional survey sample) was 30%, and at the age of 26 years (the median age of the app sample), it was 62%. The CMS substantially overrepresented never-married women by 11% (1004/2464, 40.75%); the app sample (6189/10,244, 60.42% never married) was comparable with the benchmark.

The app and CMS samples were similar in ethnicity and race composition, and each sample was reasonably similar in the distribution of the racial and ethnic minority categories to those reported in the US census (Table 3). However, both modalities, more so the CMS, had a lower percentage of White respondents than did the benchmark. The CMS provider had set numeric quotas for each ethnicity category. Although the intended total sample size was achieved, the total for White respondents was 15% less than its preset quota. These differences in outcomes are at least partially attributable to the different approaches taken to collecting race and ethnicity data. The US census asked 2 separate questions (“race” and “ethnicity”), and the survey questionnaires asked only 1 question but allowed for multiple responses.

**Table 2:** Demographics of participants in the United States—composition of the conventional modality survey (CMS) and app-based survey samples (comprising respondents from unknown locales across the United States) compared with the composition of the US 2020 census.

		CMS survey sample (total n=2464), n (%)	App-based survey sample (total n=10,244), n (%)	Benchmark survey (US 2020 census; %^a^)
**Age (years)**				
	18-19	128 (5.19)	1036 (10.11)	7
	20-24	340 (13.8)	3395 (33.14)^b^	17
	25-29	378 (15.34)	2526 (24.66)^b^	18
	30-34	492 (19.97)	1794 (17.51)	19
	35-45	1126 (45.7)^b^	1493 (14.57)^c^	39
	Median (1st quartile-3rd quartile)	33.0 (27-39)	26.0 (22-31)	—^d^
**Educational level**				
	No formal education or only primary education	175 (7.1)	38 (0.37)^c^	9
	Secondary (high school) or equivalent	702 (28.49)	2848 (27.8)	26.5
	Tertiary (at least some postsecondary education)	1587 (64.41)	7340 (71.65)^b^	63
	No answer	0 (0)	18 (0.18)	1.5
**Locale**				
	Rural	409 (16.6)	1243 (12.13)	14
	Suburban	1195 (48.5)^c^	4523 (44.15)^c^	55
	Urban	860 (34.9)	4464 (43.58)^b^	31
	No answer	0 (0)	14 (0.14)	0
**Relationship status**				
	Never married	1004 (40.75)^b^	6189 (60.42)	62 at the age of 26 years; 30 at the age of 33 years
	Married or engaged	1202 (48.78)^c^	3621 (35.35)	—
	Divorced or separated	258 (10.47)^c^	421 (4.11)	—
	No answer	0 (0)	13 (0.13)	—

^a^Percentage of those aged 18-45 years for all demographics except education level, which is percentage of those aged ≥25 years.

^b^Overrepresentation compared with benchmark.

^c^Underrepresentation compared with benchmark.

^d^Not available.

**Table 3:** Race or ethnicity of participants in the United States—composition of the conventional modality survey (CMS) and app-based survey samples (comprising respondents from unknown locales across the United States) compared with the composition of the US 2020 census.

Selected response	CMS sample (n=2769^a^), n (%)	App-based sample (n=11,676^a^), n (%)	US 2020 census of the population (%)
Asian	248 (9)^b^	698 (6)^b^	5.9^c^
Black	392 (14.2)^b^	1040 (8.9)^b^	13.4^c^
Hawaiian or Pacific Island Native	22 (0.8)^b^	49 (0.4)^b^	0.3^c^
Hispanic	550 (19.9)^b^	1884 (16.1)^b^	18.9^d^
American Indian or Alaska Native	76 (2.7)^b^	339 (2.9)^b^	1.3^c^
White	1436 (51.9)^b^	7510 (64.3)^b^	76.3^c^
No answer or “other”	45 (1.6)	156 (1.3)	—^e^

^a^Response total is greater than the number of respondents as respondents may select more than one of the listed options. For each modality, the table shows the percentage of responses in each category.

^b^Includes those who chose (1) this category alone and (2) this category in addition to any other category.

^c^Includes those who chose (1) this category alone and (2) this category and Hispanic.

^d^Includes those who chose Hispanic and one or more of the other categories.

^e^Not available.

#### India

The CMS sample, after meeting quality controls, comprised 2515 respondents from 2 states, Bihar and Uttar Pradesh. The final app-based sample, after meeting quality controls, comprised 1246 respondents from across India.

Compared with the Indian national DHS sample (Table 4), the app sample overrepresented persons aged <30 years and underrepresented those aged ≥30 years. The disparities between the app and benchmark samples were greater for the other variables. The app sample substantially underrepresented those with less education and those belonging to historically disadvantaged groups (“caste”) [37]. Although 67.5% of Indians live in rural communities, only 3.85% (48/1246) of the app sample did. Similarly, 72% of the benchmark sample were married or engaged, but only 30.58% (381/1246) of the app sample reported this status.

**Table 4:** India participant demographics—the app-based survey sample (respondents from unknown locales across India) compared with the national Demographic and Health Survey (DHS) sample, and the conventional modality survey (CMS) sample (collected in Bihar and Uttar Pradesh) compared with the DHS samples from these same 2 states.

		App-based sample (total n=1246), n (%)	India DHS 2019-2021 (table 3.1), % of sample (weighted %)^a^	CMS sample (total n=2515), n (%)	Bihar DHS 2019-2021 (tables 3, 17), % of sample (weighted %)^a^	Uttar Pradesh DHS 2015-2016 (tables 3, 10), % of sample (weighted %)^a^
**Age (years)**						
	18-19	167 (13.4)^b^	6.8 (8.4)	72 (2.9)^c^	4.5 (11.7)	2.2 (5.7)
	20-24	518 (41.6)^b^	16.5 (20.5)	718 (28.5)^b^	8.8 (23)	9.7 (25.5)
	25-29	345 (27.7)^b^	16.2 (20.1)	837 (33.3)^b^	7.5 (19.6)	7.8 (20.5)
	30-34	146 (11.7)^c^	13.9 (17.2)	402 (16)	6.2 (16.1)	6.5 (17.1)
	35-45	70 (5.6)^c^	27.2 (33.7)	486 (19.3)^c^	11.3 (29.3)	11.9 (31.2)
	Median (1st quartile-3rd quartile)	24 (21-28)	—^d^	26 (24-32)	—	—
**Educational level**						
	No formal education	0 (0)^c^	22.6	550 (21.9)^c^	38	36
	Primary	8 (0.6)^c^	18.6 (1-7 years^e^)	1011 (40.2)^b^	33 (1-9 years^e^)	31 (1-9 years^e^)
	Secondary (high school)	200 (16.1)^c^	33 (8-11 years^e^)	704 (28)^b^	13 (10-11 years^e^)	10 (10-11 years^e^)
	Tertiary (college or above)	1031 (82.7)^b^	26 (≥12 years^e^)	250 (9.9)^c^	16 (≥12 years^e^)	23 (≥12 years^e^)
	No answer	7 (0.6)	—	0 (0)	—	—
**Locale**						
	Rural	48 (3.9)^c^	67.5	2482 (98.7)^b^	84	74
	Suburban	176 (14.1)	Not included	24 (1)	Not included	Not included
	Urban	1012 (81.2)^b^	32.5	9 (0.4)^c^	16	26
	No answer	10 (0.8)	—	0 (0)	—	—
**Relationship status**						
	Never married	837 (67.2)^b^	23.6	39 (1.6)^c^	23	29
	Married or engaged	381 (30.6)^c^	72	2457 (97.7)^b^	75	68
	Divorced or separated or widowed	20 (1.6)	1	17 (0.7)	2	3
	No answer	8 (0.6)	—	2 (0.1)	—	—
**Social group**						
	Scheduled caste	78 (6.3)^c^	21.9	848 (33.7)^b^	24	23
	Scheduled tribe	23 (1.8)^c^	9.3	160 (6.4)	4	1
	Other backward class	149 (12)^c^	42.9	1232 (49)	54	54
	None of the above (general category castes)	961 (77.1)^b^	25.2	271 (10.8)^c^	17	22
	Other or no answer	43 (3.5)	—	4 (0.2)	1	0.2

^a^Percentage of those aged 15-49 years (weighted to match this study's age categories).

^b^Overrepresentation compared with benchmarks.

^c^Underrepresentation compared with benchmarks.

^d^Not available.

^e^Years of schooling.

Compared with benchmark samples from the 2 Indian states that were surveyed in this study (Table 4), the CMS sample had a comparable percentage of those aged 30 to 34 years, overrepresented those in their 20s, and substantially underrepresented the youngest and oldest cohorts. The CMS sample underrepresented those with no formal schooling or with a college education and substantially overrepresented those with 1 to 11 years of schooling, those who were married, and those in rural communities.

In sum, compared with their respective benchmarks, the app and CMS survey samples both overrepresented those in their 20s and substantially underrepresented those aged ≥35 years. The app sample overrepresented those aged 18 to 19 years; the CMS sample underrepresented this cohort. Both modalities underrepresented those without formal education; the app sample dramatically overrepresented those who had at least some higher education.

#### South Africa

Compared with the South African national DHS (the benchmark; Table 5), the CMS overrepresented those aged 25 to 29 years and underrepresented those aged 35 to 45 years. In contrast, the app sample overrepresented those aged <25 years and substantially underrepresented those aged ≥30 years. The CMS underrepresented those with a primary education and overrepresented those with secondary education. The app sample substantially overrepresented those with postsecondary education and underrepresented those with less education.

Compared with the benchmark, the CMS and app samples overrepresented never-married persons. The CMS also overrepresented married or engaged persons and urban residents. However, these discrepancies likely reflect, at least to some extent, the differences between these instruments and the response options used in the South African DHS, which did not include “suburban” as an option for locale but did include “living together” as an option for relationship status.

The ethnicity or race distribution (referred to as “population group” in the South African DHS) in the CMS was comparable with that of the benchmark. In the app sample, Black African individuals were severely underrepresented, and all other groups were overrepresented, especially White individuals.

**Table 5:** South Africa participant demographics—composition of the aggregated conventional modality survey (CMS) sample and the app-based sample (respondents from unknown locales across South Africa) compared with the 2016 national Demographic and Health Survey (DHS).

		Aggregated CMS sample (total n=2523), n (%)	App-based sample (total n=879), n (%)	South Africa DHS 2016 (table 3.1), % of sample (weighted %)^a^
**Age (years)**				
	18-19	75 (3)	209 (23.8)^b^	3.4 (4.3)
	20-24	559 (22.2)	303 (34.5)^b^	16.6 (21.2)
	25-29	687 (27.2)^b^	179 (20.4)	17 (21.7)
	30-34	566 (22.4)	110 (12.5)^c^	15.7 (20)
	35-45	636 (25.2)^c^	78 (8.9)^c^	25.7 (32.8)
	Median (1st quartile-3rd quartile)	29.0 (24-35)	23.0 (20-28)	—^d^
**Educational level**				
	No formal education	39 (1.5)	0 (0)^c^	2
	Primary education	67 (2.7)^c^	9 (1)^c^	9.1
	Secondary (high school)	2184 (86.6)^b^	340 (38.7)^c^	77.1
	Tertiary (college or above)	233 (9.2)	527 (60)^b^	11.8
	No answer	0 (0)	3 (0.3)	0
**Locale**				
	Rural	1081 (42.8)^b^	54 (6.1)^c^	32.7 (“not urban”)
	Suburban	0 (0)	453 (51.5)^b^	Not included
	Urban	1442 (57.2)^c^	369 (42)^c^	67.3
	No answer	0 (0)	3 (0.3)	—
**Relationship status**				
	Never married	1648 (65.3)^b^	662 (75.3)^b^	58.5
	“Living together”	Not included	Not included	12.5
	Married or engaged	779 (30.9)^b^	192 (21.8)	23.3
	Divorced or separated or widowed	96 (3.8)	20 (2.3)	5.6
	No answer	0 (0)	5 (0.6)	—
**Population group**				
	Black African	2301 (91.2)	254 (28.9)^c^	86.8
	Colored	128 (5.1)	97 (11)	8.6
	Indian or Asian	38 (1.5)	47 (5.3)	1.5
	White	51 (2)	484 (55.1)^b^	3.1
	Other or no answer	6 (0.2)	34 (3.9)	0.1

^a^Percentage of those aged 15-49 years (weighted to match this study's age categories).

^b^Overrepresentation compared with benchmark.

^c^Underrepresentation compared with benchmark.

^d^Not available.

### Contraceptive Use

#### United States

The app and CMS samples had similar proportions of current contraceptive users (7748/10,244, 75.63% and 1778/2464, 72.16%, respectively), which were much higher than that of the US benchmark sample (47%; Table 6). Of these current contraceptive users, the CMS and benchmark respondents had similar proportions of hormonal (approximately two-thirds; 1184/1778, 66.59% and 60%, respectively) and nonhormonal (approximately one-third; 570/1778, 32.06% and 29%, respectively) method users. In contrast, the app sample had more nonhormonal than hormonal method users (4147/7748, 53.52% vs 3033/7748, 39.15%), principally because of the lower use of the pill and higher use of nonhormonal methods than in the CMS and benchmark samples.

**Table 6:** Current contraceptive use in the United States—conventional modality survey (CMS) and app-based samples compared with the 2020 US National Survey of Family Growth (NSFG) 2017 to 2019 [38].

Responses					CMS sample (n=2464), n (%)		App-based sample (n=10,244), n (%)		NSFG (aged 15-49 years, % of sample)
**Current contraceptive users**					1778 (72.2^a,b^)		7748 (75.6^a,b^)		Weighted %^c^ (47^a^)
	**Hormonal methods (total)**			1184 (66.6^c^)		3033 (39.1^c,d^)		60^c^ (28.4^a^)	
		**LARC^e^ methods**		421 (23.7^c^)		1216 (15.7^c^)		26^c^ (12.4^a^)	
			IUD^f^ or IUS^g^	192 (10.8^c,d^)		827 (10.7^c,d^)		18^c^ (8.4^a^)	
			Injection	143 (8^c^)		43 (0.6^c^)		4^c^ (2^a^)	
			Implant	86 (4.8^c^)		346 (4.5^c^)		4^c^ (2^a^)	
		**Non-LARC methods**		763 (42.9^c^)		1757 (22.5^c^)		34^c^ (16^a^)	
			Pill	763 (42.9^b,c^)		1586 (20.5^c,d^)		30^c^ (14^a^)	
			Ring or patch	0 (0)		171 (2.2^c^)		4^c^ (2^a^)	
	Morning-after pill			24 (1.3^c^)		60 (0.8^c^)		N/A^h^	
	**Nonhormonal methods (total)**			570 (32.1^c^)		4147 (53.5^b,c^)		29^c^ (13.8^a^)	
		Condoms		354 (19.9)^c^		1894 (24.4)^c^		18^c^ (8.4^a^)	
		Natural methods		128 (7.2)^c^		1598 (20.6)^b,c^		11^c^ (5.4^a^)	
		Copper IUD		31 (1.7)^c^		655 (8.5)^c^		0 (0)	
		Other methods		57 (3.2^c,d^)		568 (7.3)^c,d^		12^c^ (5.6^a^)	
**Other responses**					686 (27.8^a^)		2496 (24.4^a^)		53^a^
	None used in the last 3 months			510 (20.7^a,d^)		1688 (16.5)^a,d^		34.7^a^	
	Female sterilization			N/A		N/A		18^a^	
	No answer or N/A			176 (7.1^a^)		808 (7.9^a^)		N/A	

^a^Percentage of survey sample.

^b^Overrepresentation compared with benchmark.

^c^Percentage of current contraceptive users (samples do not include sterilized respondents).

^d^Underrepresentation compared with benchmark.

^e^LARC: long-acting reversible contraception.

^f^IUD: intrauterine device.

^g^IUS: intrauterine system.

^h^N/A: not applicable.

#### India

The rates of current contraceptive use (Table 7) in the benchmark samples were much lower (24.5% in the DHS Bihar/Uttar Pradesh sample and 29% in the DHS national sample) than those in the Bihar/Uttar Pradesh CMS sample (2217/2515, 88.15%) or the nationwide app sample (637/1246, 51.12%). Nonetheless, in each sample, the proportion of current contraceptive users relying on nonhormonal methods was clearly the highest proportion of such users and approximately similar (71%-88%) across all 4 samples. App respondents were approximately twice as likely to use condoms (387/637, 60.8%) as the other samples, a practice that largely accounts for the high reliance on nonhormonal methods in the app sample. More than half (56%) of the Bihar/Uttar Pradesh DHS sample relied on nonhormonal methods; only 27.5% (610/2217) relied on these methods in the Bihar/Uttar Pradesh CMS sample.

Half of the Bihar/Uttar Pradesh DHS sample but only 6.96% (175/2515) of the CMS sample in these states reported not having used contraception during the previous 3 months. The rates of not using contraceptives were similar in the app (359/1246, 28.81%) and DHS (33%) national samples.

**Table 7:** Current contraceptive use in India—conventional modality survey (CMS) sample (collected in Bihar: n=1234; Uttar Pradesh: n=1281) compared with Demographic and Health Survey (DHS) aggregated samples from these same 2 states (aged 15-49 years and currently married) and app-based sample (respondents from unknown locales across India) compared with 2019 to 2021 national DHS sample (figure 5.1 and table 5.5 in India DHS report; aged 15-49 years and currently married).

Responses							Bihar and Uttar Pradesh									National				
							CMS sample (n=2515), n (%)				DHS (Bihar and Uttar Pradesh, approximately n=98,000)^a^				App-based sample (n=1246), n (%)					DHS 2019-2021 (approximately n=521,300)
**Current users**							2217 (88.2)^b,c^				24.5^b^				637 (51.1)^b,c^					29^b^
	**Hormonal methods (total)**					581 (26.2)^c,d^				15.6^d^				76 (11.9)^d,e^					27.3^d^	
		**LARC^f^ methods**			176 (7.9)^d^				7.4^d^				3 (0.5)^d^					9^d^		
			IUD^g^ or IUS^h^	0				4.2^d^				3 (0.5)^d,e^					7^d^ (2.1^b^)			
			Injection	176 (7.9)^d^				3.2^d^				0					2^d^ (0.6^b^)			
			Implant	0				0				0					0			
		**Non-LARC methods**			395 (17.8)^d^				8.2^d^				48 (7.5)					18.3^d^		
			Pill	395 (17.8)^c,d^				8.2^d^				44 (6.9)^d,e^					18^d^ (5.1^b^)			
			Patch or Ring	0				0				4 (0.6)^d^					0			
	Morning-after pill				10 (0.5)^d^				0				25 (3.9)^d^					0.3^d^ (0.1^d^)		
	**Nonhormonal methods (total)**					1636 (73.8)^d,e^				85^d^				560 (87.9)^c,d^					71^d^	
		Condoms			777 (35)^d^				29^d^				387 (60.8)^c,d^					33^d^ (9.5^b^)		
		Natural methods			610 (27.5)^d,e^				56^d^				151 (23.7)^d,e^					38^d^ (11^b^)		
		Copper IUD^g^			76 (3.4)^d^				0				9 (1.4)^d^					0		
		Other methods				173 (7.8)^d^				0				13 (2)^d^					1^d^ (0.3^b^)	
**Other responses**							298 (11.8)^b^				75.5^b^				609 (48.9)^b^					71^b^
	None used in last 3 months					175 (7)^b,e^				49.5^b^				359 (28.8)^b^					33^b^	
	Female sterilization					N/A^i^				26^b^				N/A					38^b^	
	No answer or N/A					123 (4.9)^b^				N/A				250 (20.1)^b^					N/A	

^a^Equal weighting of Bihar and Uttar Pradesh.

^b^Percentage of survey sample.

^c^Overrepresentation compared with benchmark.

^d^Percentage of current contraceptive users (samples do not include sterilized respondents).

^e^Underrepresentation compared with benchmark.

^f^LARC: long-acting reversible contraception.

^g^IUD: intrauterine device.

^h^IUS: intrauterine system

^i^N/A: not applicable.

#### South Africa

The CMS and benchmark samples (Table 8) had similar rates of current contraceptive use (1130/2523, 44.79% and 47%, respectively), and the app sample overrepresented current users (586/879, 66.7%).

However, the CMS sample underrepresented hormonal methods compared with the benchmark (703/1130, 62.21% vs 73%, respectively), especially LARC methods (519/1130, 45.93% vs 62%, respectively). Among those using hormonal contraception in the CMS and benchmark samples, the respective rates of use of the pill (139/1130, 12.3% and 11%) and intrauterine devices (11/1130, 0.97% and 2%) were similar.

**Table 8:** Current contraceptive use in South Africa—conventional modality survey (CMS) and app-based samples compared with 2016 Demographic and Health Survey (DHS) sample (aged 15-44 years).

Responses						CMS sample (n=2523), n (%)			App sample (n=879), n (%)			DHS 2016 (n=7632)
**Current users**						1130 (44.8)^a^			586 (66.7)^a,b^			Weighted %^c^ (47^a^)
	**Hormonal methods (total)**					703 (62.2)^c,d^			203 (34.6)^c^			73^c^
		**LARC^e^ methods**			519 (45.9)^c^			56 (9.6)^c^			62^c^	
			IUD^f^ or IUS^g^ (coil)	11 (1)^c^			33 (5.6)^c^			2^c^ (1^a^)		
			Injection	411 (36.4)^c^			14 (2.4)^c^			53^c^ (24^a^)		
			Implant	97 (8.6)^c^			9 (1.5)^c^			7^c^ (3^a^)		
		**Non-LARC methods**			139 (12.3)^c^			133 (22.7)^c^			11^c^	
			Pill	139 (12.3)^c^			125 (21.3)^b,c^			11^c^ (5^a^)		
			Patch or Ring	0 (0)			8 (1.4)^c^			0 (0)		
	Morning-after pill				45 (4)^c^			14 (2.4)^c^			<1^c^ (<1^a^)	
	**Nonhormonal methods (total)**					427 (37.8)^b,c^			289 (49.3)^b,c^			27^c^
		Condoms			379 (33.5)^b,c^			192 (32.8)^b,c^			26^c^ (12^a^)	
		Natural methods			48 (4.2)^c^			26 (4.4)^c^			<1^c^ (<1^a^)	
		Copper IUD			0 (0)			39 (6.7)^c^			0 (0)	
		Other methods				0 (0)			32 (5.5)^c^			<1^c^ (<1^a^)
**Other responses**						1393 (55.2)^a^			293 (33.3)^a^			53^a^
	None used in last 3 months					1393 (55.2)^a^			196 (22.3)^a^			50^a^
	Female sterilization					N/A^h^			N/A			3^a^
	No answer or N/A					0 (0)			97 (11)^a^			N/A

^a^Percentage of survey sample.

^b^Overrepresentation compared with benchmark.

^c^Percentage of current contraceptive users (samples do not include sterilized respondents).

^d^Underrepresentation compared with benchmark.

^e^LARC: long-acting reversible contraception.

^f^IUD: intrauterine device.

^g^IUS: intrauterine system.

^h^N/A: not applicable.

Although current contraceptive users were overrepresented in the app sample (586/879, 66.7%) relative to the benchmark, the use of hormonal methods was much lower (203/586, 34.6%). Nonetheless, compared with the benchmark, the app sample overrepresented pill users (125/586, 21.3%).

Injection was the most commonly used current method in South Africa (53%); however, only 36.37% (411/1130) of the CMS respondents and 2.4% (14/586) of the app respondents were using this method. In contrast, condoms were overrepresented in both the CMS and app samples.

### Attitudes Regarding Menstrual Bleeding

For each country, we compared the data on menstrual bleeding attitudes collected using the app survey and the CMS (Table 9). Benchmark data for these variables are not available as comparable questions have not been included in the DHS or, to our knowledge, in any other nationally representative survey study in these countries. However, menstrual bleeding attitudes, including whether people feel menstruation is a burden or a benefit (or a mix of both), can have important implications for contraceptive decision-making and overall health [39]. As MTA users have self-selected to track their menstrual cycles, ascertaining whether their perspectives on menstruation differ from that of the broader population can be important for designing research studies and intervention protocols.

**Table 9:** Menstrual bleeding attitudes—conventional modality survey (CMS) and app samples.

		United States		India		South Africa	
		CMS sample (n=2469), n (%)	App sample (n=10,244), n (%)	CMS sample (n=2515), n (%)	App sample (n=1246), n (%)	CMS sample (n=2523), n (%)	App sample (n=879), n (%)
**I need to have periods to be in good health**							
	Positive	1030 (42)	4254 (42)	2288 (91)	936 (75)	1649 (65)	472 (54)
	Neutral	683 (28)	2455) (24)	100 (4)	131 (11)	325 (13)	192 (22)
	Negative	756 (31)	3530 (34)	46 (2)	173 (14)	549 (22)	213 (24)
	No answer	0 (0)	5 (<0.5)	81 (3)	6 (<0.5)	0 (0)	2 (<0.5)
**I wish I could take a break from having my period**							
	Positive	1570 (64)	6842 (67)	1054 (42)	639 (51)	1124 (45)	551 (63)
	Neutral	506 (20)	1773 (17)	550 (22)	236 (19)	283 (11)	151 (17)
	Negative	393 (16)	1624 (16)	830 (33)	364 (29)	1116 (44)	175 (20)
	No answer	0 (0)	5 (<0.5)	81 (3)	7 (1)	0 (0)	2 (<0.5)
**I don’t want to change my natural menstrual cycle**							
	Positive	1036 (42)	4727 (46)	1110 (44)	1003 (80)	1638 (65)	515 (59)
	Neutral	747 (30)	2073 (20)	489 (19)	101 (8)	302 (12)	156 (18)
	Negative	686 (28)	3439 (34)	835 (33)	135 (11)	583 (23)	206 (23)
	No answer	0 (0)	5 (<0.5)	81 (3)	7 (1)	0 (0)	2 (<0.5)
**How often would you like to have your period?**							
	Regular periods	953 (43)^a^	4265 (42)	2169 (86)	811 (65)	1344 (53)	448 (51)
	Regular periods but able to skip sometimes	586 (26)^a^	2241 (22)	135 (5)	207 (17)	605 (24)	185 (21)
	Not have periods except when I want to become pregnant	301 (14)^a^	1752 (17)	77 (3)	123 (10)	349 (14)	131 (15)
	Never have a period	381 (17)^a^	1973 (19)	53 (2)	102 (8)	225 (9)	111 (13)
	No answer	248 (10)	13 (<0.5)	81 (3)	3 (<0.5)	0 (0)	4 (<0.5)
**Who would you be comfortable talking to about your period?** **(check all that apply)**							
	Anyone	566 (23)	3878 (38)	177 (7)	440 (35)	521 (21)	320 (36)
	Some or all friends	1373 (56)	9275 (91)	488 (19)	1034 (83)	448 (18)	744 (85)
	Family members	1278 (52)	7891 (77)	851 (34)	901 (72)	380 (15)	633 (72)
	My partner or partners	1396 (57)	9152 (89)	2033 (81)	1115 (89)	640 (25)	759 (86)
	Physician or nurse	1665 (67)	9764 (95)	1178 (47)	1071 (86)	977 (39)	779 (89)
	Pharmacist	791 (32)	6201 (61)	160 (6)	574 (46)	182 (7)	554 (63)
	Health worker	770 (31)	7939 (77)	347 (14)	791 (63)	410 (16)	631 (72)
	None of these	327 (13)	91 (1)	95 (4)	27 (2)	107 (4)	14 (2)

^a^Percentage of all those (n=2221) who answered this question.

In the United States, the app and CMS samples had similar distributions of responses to the first 4 questions. Pluralities agreed that periods are necessary for good health, that they did not want to change their natural menstrual cycle, and that they wanted regular periods. However, a clear majority of each sample (CMS: 1570/2469, 64%; app: 6842/10,244, 67%) also agreed with the following statement: “I wish I could take a break from having my period.” Notably, a much larger proportion of the Clue app sample than of the CMS sample was comfortable talking about their periods with a variety of people (medical personnel, partners, friends, and family). Only somewhat more than half of the CMS sample was comfortable speaking to these categories of persons; less than a third were comfortable speaking to pharmacists and health care workers.

In India, most of the CMS and app samples agreed that periods are necessary for good health and wanted regular periods (Table 9). However, the CMS sample was more likely to prefer to have regular periods (86%), and only 42% selected that they wanted to take a break from their periods compared with 65% and 51%, respectively, for the app sample. In contrast, most (80%) of the app sample selected that they did not want to change their natural cycle compared with only 44% of the CMS sample. A high percentage of the national sample of app users were open to speaking about their period with medical staff, partners, friends, and family; slightly less than half were comfortable speaking to pharmacists. The CMS conducted in Bihar and Uttar Pradesh found high comfort in respondents regarding speaking with their husbands but much less comfort speaking with anyone else.

In South Africa, the app and CMS samples were very similar in the distributions of preferred frequency of menstrual bleeding and maintaining the natural menstrual cycle (majorities wanted regular periods and did not want to change their cycles). Most of both South African samples also agreed that periods are needed to be in good health, although the proportion of those who agreed with this opinion was higher in the CMS sample. The app sample was much more likely to want a break from periods than the CMS sample (65% vs 45%). High proportions of the app sample were comfortable talking about their periods with medical staff, partners, and friends; a smaller but still majority percentage of app users would speak with family, health care workers, and pharmacists. Although 39% of the CMS sample were comfortable speaking to physicians and nurses, a quarter or less was comfortable speaking to anyone else.

## Discussion

### Principal Findings

#### Overview

The goal of this study was to investigate the feasibility of conducting reproductive health surveys using mainstream MTAs. The expanding use of smartphones and related apps provides a novel means of reaching large and diverse pools of potential research participants, but little is known about the suitability of using this novel modality to gather reproductive health data. In this study, we evaluated ease of use; ability to achieve desired sample sizes; and representativeness with regard to demographic variables, contraceptive use, and menstrual bleeding attitudes of the responses collected using the Clue MTA compared with responses collected using CMSs or nationally representative benchmark data in 3 target countries.

#### Performance

In the United States, the target sample size of 10,000 completed questionnaires collected via the Clue app was reached in <3 weeks, whereas targets were not met in India or South Africa, where the number of active users was lower. Similar trends were observed for the use of CMSs. Although the target sample size was quickly met using the web-based CMS in the United States, the SMS text messaging–based CMS in South Africa was not able to meet the target quotas and required supplementation using another method (in-person tablet surveys). In India, in-person interviews were able to meet the desired sample size, but because of COVID-19 pandemic challenges, the protocols and time frame had to be adjusted to do so.

Thus, when user numbers were sufficient, app-distributed surveys were able to quickly capture large samples on par with other methods and at low cost. An additional advantage of app distribution, as with other digital platforms with a global reach, was that recruitment could be deployed remotely and simultaneously across countries. In addition, respondents were able to participate from the safety and comfort of the location of their choosing, which enhanced ease of participation during the COVID-19 pandemic.

#### Demographic Variables

In each of the 3 countries, there were clear demographic differences between the samples of the different modalities, with neither the app samples nor the CMS samples emerging as a consistently better match to the distribution of attributes observed in the respective national-level benchmarks. Despite the efforts to achieve reasonable demographic representativeness in the CMS samples, it is notable that this goal was not achieved. The CMS samples deviated from the benchmarks in country-specific ways, sometimes over- and other times underrepresenting demographic subgroups. For example, the CMS sample underrepresented married individuals in the United States, overrepresented married individuals and rural residents in India (to the near exclusion of any other locales or relationship statuses), and skewed toward midrange age groups in South Africa.

When comparing the app users to the respective benchmarks across all countries, the app samples consistently overrepresented those who were younger, were urban residents, and had more education. In particular, postsecondary education was higher in the app user group than in the broader population, although this disparity was more marked in India and South Africa than in the United States. These patterns are consistent with younger, more educated persons being more likely to own a smartphone and use health apps [10,40]. In India and South Africa, the app samples also overrepresented never-married persons from more socially or economically advantaged groups, whereas in the US app sample, relationship status and ethnicity or race representation were more comparable with the benchmark.

Of the 3 countries, app users in the United States were the most diverse sample, likely a result of the larger user base and higher market penetration. In the United States, it is estimated that one-third of women use MTAs [11]. Although MTA use is common worldwide, users in countries with more limited smartphone and data access such as India and South Africa may be more representative of more advantaged early adopters of the technology, a group that may have unique characteristics. However, as current trends continue and the gender and socioeconomic divide narrows with regard to access to technology, these differences will lessen.

It should also be noted that, for any platform, the user base is reflective of the app’s current features and the company’s marketing strategies. For example, when this study was conducted, the primary functionality of the Clue app was general period tracking. However, with additional features such as potential modes for trying to conceive or tracking perimenopause symptoms, the expected user base would be significantly different. Thus, platform functionality and other factors that could influence user base characteristics should be evaluated when selecting a research platform and developing strategies intended to achieve study samples that are suitable for the specific research questions.

#### Current Contraceptive Use

The app samples in all 3 countries had substantially higher proportions of current contraceptive users than the respective benchmark samples. In the CMSs, the United States and India also had higher proportions of current contraceptive users, whereas the South African sample was similar to the benchmark.

Although the proportion of contraceptive types varied across survey modalities, in all 3 countries, the app sample overrepresented nonhormonal method users. This is to be expected as, at the time of the study, the Clue app was most commonly used for menstrual tracking and period predictions. Thus, those who do not have menstrual periods (because of, for example, hormonal contraceptive–induced amenorrhea or hormonal regimens that produce predictable periods) may be less likely to find value in such an app and, therefore, are less likely to be represented in the data sample. Although Clue does have features for hormonal contraceptive users, such as contraceptive tracking and reminders, these are less frequently used than features for tracking monthly bleeding by nonhormonal contraceptive users.

The CMS samples deviated from the benchmarks in ways that are likely reflective of the unique circumstances (ie, the COVID-19 pandemic) at the time of the research. For example, in the CMS sample in India, there was a higher current use of contraceptives, including hormonal methods, compared with the benchmark. In this instance, the CMS interviews were conducted by local health workers (with extra safety protocols in place because of the COVID-19 pandemic). The refusal rates to participate were much higher than in typical research in the area, likely because of pandemic fears. In addition, there were local initiatives to increase access to contraceptives, including long-acting injectables, during the pandemic. Women who agreed to participate in the research may have been more comfortable with local health workers and local initiatives, potentially biasing the sample toward certain characteristics. Thus, a comprehensive evaluation of the sample and external factors that could influence research results should always be considered during research planning and data interpretation regardless of method.

#### Menstrual Bleeding Attitudes

Given the specific purpose of MTAs for tracking menstrual cycles, it was anticipated that these app users could differ from the broader population with regard to attitudes toward menstrual bleeding (such as whether they wanted to have regular cycles vs preferring cessation of menstruation or whether they felt they needed to have periods to be in good health), which led to self-selection for using MTAs. However, in each of the 3 countries, bleeding attitudes from the respective app survey and CMS samples were reasonably similar.

The one exception was for the following question: “Who would you be comfortable talking to about your period?” Although CMS respondents in all countries were generally not comfortable discussing their periods with a wide array of others, most Clue app respondents were comfortable talking with their partners, friends, family, and health care providers. Furthermore, there was little difference between the Clue users’ responses across countries.

These patterns suggest that, although attitudes toward menstruation are generally similar between MTA users and the general population, app users may be more receptive to responding to questions on sensitive topics regarding women’s health. In addition, having an interest in the subject matter (demonstrated by their interest in tracking aspects of their own health) indicates that app users may be more invested, compared with a general audience, in contributing to research on a personally meaningful topic. Furthermore, entering responses via a smartphone app may be perceived as more private than some CMS methods, a factor that may prompt MTA respondents to answer questions regarding reproductive health more truthfully and comprehensively.

### Study Limitations

Different CMSs and strategies were used to optimize the CMS protocols in each of the 3 countries. These decisions were based on several factors, including (most importantly) local experts’ advice on which modalities could be expected to achieve a sufficient sample of diverse respondents at a low cost. Although the same base survey questions were used for all CMSs, different modifications were used to meet the requirements of each. For example, the SMS text message questions and answers in South Africa had to be modified to fit very low limits on the number of characters that could be used. This modification was acceptable for the goals of this study, but these differences in wording may limit the comparability of CMS findings across the 3 countries.

In addition, this research was conducted during the COVID-19 pandemic, which affected data collection. Some protocols were adjusted, such as the modification of interview protocols in India to ensure the safety of participants and interviewers, and some timelines for data collection were shifted, again, particularly in India as the interviews were conducted face-to-face. These unavoidable changes resulted in slightly different time frames for data collection. As the impact of the pandemic also varied by country and time frame, variables such as willingness to participate, current contraceptive methods used, and the resulting needs and preferences of respondents were differentially affected. These factors further limited the direct comparability of CMS data across samples and countries.

When evaluating the Clue samples specifically, there was likely selection bias in who chose to participate in surveys sent via the app. As demographic information is not routinely collected by the Clue app, it is not possible to ascertain whether these survey participants were a reasonable representation, at the time of data collection, of the overall Clue user base. In addition, the smaller size of the Clue samples from India and South Africa may have limited our ability to draw conclusions about app users in these countries. However, as with any commercial platform, user demographic characteristics are only a snapshot of the current user population. As a user base likely changes over time, the specific demographic profile of any research sample is best assessed concurrently with the collection of the data of principal interest in a given study.

### Conclusions

This study found that MTAs with a sufficiently large user base provide a quick and inexpensive means of reaching potential participants for reproductive health research. In all 3 target countries, Clue app survey respondents were younger, more educated, more likely to be urban residents, and more likely to use nonhormonal contraceptive methods than the national benchmark samples. Within each country, the app-based and CMS samples expressed comparable attitudes toward menstruation. However, MTA users were more comfortable discussing their menstrual cycles with others, which suggests that MTA users may be more inclined to respond truthfully and fully to sensitive reproductive health questions.

These demographic and attitudinal consistencies in the data, as well as the ability to distribute surveys remotely and simultaneously across countries using MTAs, lends strength to cross-country comparisons of app-based survey findings. In contrast, CMS samples deviated in country-specific ways, sometimes over- and other times underrepresenting demographic and contraceptive use subgroups compared with the benchmark samples. Thus, regardless of the research method, it is important to define the desired participant sample and collect sociodemographic data to confirm the composition of the obtained sample. Nonetheless, in choosing a research modality, factors such as cost, target sample size, or the need for a specific target group may be more important than demographic representativeness. A benefit of mainstream MTAs for reproductive health data collection are the large-scale, globally distributed user groups with a heightened interest in this aspect of health and who likely have greater comfort in discussing these and related (often taboo) issues. Thus, apps such as Clue offer an alternative platform for data collection and an exciting opportunity to understand the role of population-specific sociocultural beliefs and practices in achieving satisfactory reproductive health for all.

## Appendix

Multimedia Appendix 1 Conventional modality survey methods for each study country.

## Abbreviations

CMS: conventional modality survey
DHS: Demographic and Health Survey
mHealth: mobile health
MTA: menstrual tracking app

## Data Availability

The deidentified data sets used and analyzed during this study are available at the Dryad Digital Repository [39].
