# Supplementary material for: Assessment of App-Based Versus Conventional Survey Modalities for Reproductive Health Research in India, South Africa, and the United States: Comparative Cross-Sectional Study
Source: JMIR Form Res. 2023 Dec 1;7:e44705. doi: 10.2196/44705 (PMC10724813; doi:10.2196/44705)
Supplement: Multimedia Appendix 1 [file formative_v7i1e44705_app1.docx]

**Multimedia Appendix 1. Conventional Modality Survey Methods for each Study Country**

***United States***

In the US, Dynata [29], a market research company, administered the questionnaire via a website, considered to be the approach most likely to access potential US participants across a wide range of sociodemographic characteristics. So as to reasonably match the general US population, Dynata set maximum quotas for two demographic variables: ethnicity/race (White=1700; Black=425; Latinx=650; Asian=250; Other=100) and education (no formal education/some formal education/primary education=275; high school education=700; college degree or diploma=1750; postgraduate=400).

The questionnaire was device agnostic (i.e., could be taken on computer or mobile devices). No personal identifiable information was collected. Because of length and cost constraints, the questionnaire did not include a quality check question. The time to complete the questionnaire was less than 20 minutes. The sample target (total N = 2500) was reached in less than three weeks (November 6-24, 2020).

***India***

Data collection in India was conducted by Sigma [30], a research and consulting organisation for the social and development sector. Two states, Uttar Pradesh and Bihar, were selected for the survey because of their relatively high proportion of low income residents. Sigma aimed to use probabilistic sampling to achieve representativeness in the study sample.

Local health care workers verbally administered the questionnaire during in-person interviews. COVID-19 safety protocols included wearing masks, using sanitiser, and maintaining physical distancing, and field staff were also given medical insurance. In-person data collection was started on April 20, 2021 and continued until May 7, 2021, when the target was exceeded (total N=2515).

***South Africa***

Data collection in South Africa was conducted by GeoPoll [31] from November 6 through December 3, 2020 and by M-Ploy [32] from January 20^th^ to 26^th^, 2021. GeoPoll is a provider of remote, mobile-based research solutions throughout Africa, Asia, and Latin America. M-Ploy is a provider of fieldwork management services for market research and social research projects, and was better positioned to access lower income persons.

Individual socioeconomic status (SES) was evaluated using the 2004 Pan-African Living Standards Measure (LSM), a widely used standard for evaluating SES throughout Africa [33], [34]. Classification is based on ownership of a standard list of goods (e.g., desktop computers, motor vehicles, electric stoves). The LSM is preferred over household monetary income, which can be unstable and difficult to report accurately.

GeoPoll administered the questionnaire via SMS (text messaging) to mobile phones. The SMS format necessitated some questionnaire reformatting (primarily shortening of questions and responses to fit character limits). Because SMS does not require more expensive smartphones or internet access, this modality is more likely to reach an economically diverse pool of potential South African respondents. Quotas were set for a total of 2500 participants across three LSM groups, but completed questionnaires fell short of the target in the poorest category (targets followed by achieved samples in brackets: LSM 1-4 = 400 [66]; LSM 5-7 = 1700 [1700]; LSM 8-10 = 400 [400]; total N=2166).

To increase the sample size for the lowest LSM group, M-Ploy was contracted to administer in-person tablet-based questionnaires (completed by either the respondent and/or the interviewer). Target communities were randomly selected from metropolitan and rural areas; a starting point was randomly selected within each community. Female interviewers, accompanied by male escorts for security, selected respondents following a left-hand rule (if the randomly selected household did not participate, the household to its left was asked to participate) until M-Ploy reached its target sample size (n= ≥360) for LSM 1-4. A total of 357 respondents met the quality checks and the criteria for the lowest LSM. Although GeoPoll and M-Ploy survey instruments solicitated comparable data, M-Ploy used a different methodology, format, and codebook than did GeoPoll, thus necessitating additional analytical programming and data analyses, and accompanying increases in costs. The total sample size for the South African conventional survey was 2523 qualified respondents (Geopoll=2166 + Mploy=357). The totals by SES categories were LSM 1-4 = 423, LSM 5-7 =1700, and LSM 8-10 = 400.
